# Air and surface sampling for clade Ib monkeypox virus in United Kingdom hospitals, 2024 to 2025

**DOI:** 10.2807/1560-7917.ES.2025.30.19.2500288

**Published:** 2025-05-15

**Authors:** Barry Atkinson, Susan Gould, Ian Nicholls, Khanzadi Nazneen Manzoor, Jack Smith, Andrew J. Hindle, Anne J. Tunbridge, Joby Cole, Paul Collini, Alejandra Alonso, Geraldine O’Hara, Cecilia Tuudah, Jonathan A. Otter, Berkin Hack, Caroline Taylor, Thomas Pottage, Tom Fletcher, Jake Dunning

**Affiliations:** 1 Diagnostics and Pathogen Characterisation, UK Health Security Agency, Porton Down, Salisbury, United Kingdom; 2 NIHR Health Protection Research Unit in Emerging and Zoonotic Infections, Liverpool, United Kingdom; 3 Department of Clinical Sciences, Liverpool School of Tropical Medicine, Liverpool, United Kingdom; 4 Tropical and Infectious Disease Unit, Liverpool University Hospitals NHS Foundation Trust, Liverpool, United Kingdom; 5 Department of Infectious Diseases and Tropical Medicine, Sheffield Teaching Hospitals NHS Foundation Trust, Sheffield, United Kingdom; 6 Clinical Infection Research Group, Division of Clinical Medicine, School of Medicine and Population Health, University of Sheffield, Sheffield, United Kingdom; 7 Evelina London Children's Hospital, London, United Kingdom; 8 Directorate of Infection, Guy's and St Thomas' Hospital NHS Foundation Trust, London, United Kingdom; 9 Royal Free London NHS Foundation Trust, London, United Kingdom; 10 Pandemic Sciences Institute, University of Oxford, Oxford, United Kingdom

**Keywords:** Monkeypox virus, MPXV, clade Ib, environmental sampling, infection prevention and control

## Abstract

Air and surface sampling was performed in isolation rooms of seven patients with clade Ib mpox admitted to high consequence infectious disease centres in the United Kingdom. We detected monkeypox virus (MPXV) DNA in 66/90 surfaces samples and 1/14 air samples; replication competent MPXV was identified in 4/21 surface samples selected for viral isolation. These findings demonstrate that viable clade Ib MPXV contamination can occur during treatment of clade Ib mpox patients reinforcing the importance of infection prevention and control measures.

Between late October 2024 and the end of January 2025, the first eight patients with clinical symptoms of clade Ib monkeypox virus (MPXV) infection were identified in the United Kingdom (UK). Seven of them were admitted for clinical observation and monitoring to airborne high consequence infectious disease (HCID) centres. To understand if the immediate environment of patients with clade Ib mpox can become contaminated with MPXV, we investigated whether this virus could be detected in environmental surface and air samples collected from the seven patients’ rooms or anterooms.

## Case information

All seven admitted individuals were aged ≤ 60 years, with four being of male and three of female sex. Among them, four individuals had travel links with known endemic regions for clade Ib MPXV, and three individuals became infected through household transmission within the UK. Key information relating to the seven cases admitted to an HCID centre is shown in Table 1; a separate report has summarised clinical features of the first five cases [1].

**Table 1:** Information relating to clade Ib mpox cases treated in HCID centres and reported in this study, United Kingdom, 2024−2025 (n = 7 cases)^a^

Characteristic	Case 1^b^	Case 2^b^	Case 3^b^	Case 4^b^	Case 5^b^	Case 6	Case 8
Sex	Male	Female	Female	Female	Male	Male	Male
Age range in years	31–45	31–45	0–15	0–15	16–30	46–60	16–30
Transmission	Travel	Household	Household	Household	Travel	Travel	Travel
WHO clinical severity^b^	Moderate	Mild	Mild	Mild	Severe	Moderate	Severe
Recovery	Full	Full	Full	Full	Full	Full	Full

HCID: high consequence infectious disease; UK: United Kingdom; WHO: World Health Organization.

^a^ One of the eight clade Ib mpox cases identified in the UK (Case 7) was not treated in an HCID centre. The remaining seven cases described in the table were treated in either of three different HCID centres in the UK.

^b^ Some characteristics of this case have been described in a previous study [1].

^c^ Severity was rated according to WHO ‘Clinical management and infection prevention and control for monkeypox: Interim rapid response guidance, 10 June 2022’ [4].

## Environmental sampling in isolation rooms

Environmental air and surface sampling was conducted as previously described [2]. Briefly, environmental sampling was performed on seven separate occasions in rooms occupied at the time by patients with confirmed clade Ib MPXV infection (five sampling events) or ca 16 hours post discharge of patients with confirmed clade Ib MPXV infection (two sampling events). One of these sampling events took place in a room occupied by two co-habiting infected individuals (Cases 2 and 3), and two separate sampling events were performed around the same individual 6 days apart (Case 5). The sampling scheme aimed to sample the same surfaces in all rooms and to collect air samples both before and during a bed linen change; however, minor variations were made to account for different room set-ups, such as the absence of a sink in one of the patient-rooms.

Surface samples were collected using Copan flocked swabs in universal transport media and air samples were collected using a Sartorius MD8 Airport (50 L/min for 5 min). Swabbing was performed over an area approximately 10 cm × 10 cm in size where possible. Tap handles and toilet flushes were sampled in their entirety. Quantitative (q)PCR was performed using the dD14–16 assay [3].

Selected samples with detectable MPXV DNA were used for virus isolation. Samples were centrifuged at 10,000 rpm for 3 min with the supernatant diluted 1:10 in 0% Dulbecco's modified eagle medium (DMEM) culture media and used to inoculate 70% confluent monolayers of Vero E6 cells in T25 culture flasks with DMEM GlutaMAX with final concentrations of 2% fetal bovine serum, 4% antibiotic/antimycotic solution and 25 mM 4-(2-hydroxyethyl)-1-piperazineethanesulfonic acid (HEPES). Flasks were incubated at 37 °C and inspected regularly for signs of cytopathic effect, with time point samples collected to monitor MPXV DNA levels by qPCR.

## Monkeypox virus contamination levels in isolation rooms

MPXV DNA was detected in 66/90 surface samples collected (Table 2). Unsurprisingly, samples from frequently touched points often contained detectable MPXV DNA, with MPXV detected in bathroom tap handle samples collected during all seven sampling events. Similarly, the shower handle and toilet flush samples contained detectable MPXV DNA from six of seven sampling events.

**Table 2:** Findings from environmental sampling events conducted around clade Ib mpox patients treated in HCID centres, United Kingdom, 2024−2025 (n = 7 events)

Points considered	Case 1^a^	Cases 2^a^ + 3^a,b,c^	Case 4^a,c^	Case 5^a^ (visit 1^d^)	Case 5^a^ (visit 2^d^)	Case 6	Case 8
**Patient and room status at time of environmental sampling**							
Time since mpox onset	10 days	13 days (Case 2); 10 days (Case 3)	7 days	13 days	19 days	11 days	12 days
Time since admission	2 days	13 days (Case 2); 13 days (Case 3)	13 days	8 days	14 days	4 days	6 days
Time since room cleaned	24 hours	24 hours^e^	24 hours^e^	24 hours	24 hours	12 hours	24 hours
Latest patient Cq values	Day of sampling	3 days before sampling	Day before sampling	Day of sampling	Day of sampling	Day of sampling	Day of sampling
Throat	32.5	30.4 (Case 2)	ND	37.2	ND	29.5	32.2
Lesion	18.9	35.7 (Case 2)	34.1	27.2	37.2	22.5	16.4
Plasma	34.7	NA	NA	NA	NA	35.5	33.6
**Isolation room surface sample Cq values**							
Window ledge	35.2	34.5	ND	29.8	28.9	ND	**31.2**
Chair (armrest)	33.4	33.0	ND	30.2	29.6	32.9	33.0
Call button	ND	34.5	ND	25.5	32.7	37.3	ND
Light switch	**30.7**	32.2	ND	36.9	36.2	ND	34.8
Observation machine	30.6	ND	ND	28.6	34.3	34.5	34.7
Air vent	37.4	ND	ND	34.3	32.3^f^	ND	35.1
Bathroom door handle	33.2	38.0	ND	30.8	37.0	ND	31.7
Toilet flush	31.5	34.9	36.6	36.8	34.6	ND	32.5
Shower handle	33.8	37.3	ND	32.4	35.5	35.9	38.0
Tap handle (patient room)	32.5	NA	NA	29.0	29.9	32.7^g^	ND
Tap handle (bathroom)	26.4	35.2	35.9	**27.3**	27.6	32.6	34.0
TV remote / table	29.7	37.9	ND	27.2	32.0	35.6	ND
**Anteroom surface samples Cq values**							
Anteroom floor	ND	ND	ND	32.9; **26.2** ^h^	33.6	ND	33.7
**Air samples**							
Before bed linen change	ND	ND	ND	ND	ND	ND	ND
During bed linen change	ND	ND	ND	ND	35.5	ND	ND

Cq: cycle of quantification; HCID: high consequence infectious disease; NA: not applicable (not present for sampling); ND: not detected; TV: television; UK: United Kingdom.

^a^ Some characteristics of this case have been described in a previous study [1].

^b^ Cases 2 and 3 co-habited in the same HCID isolation room.

^c^ Environmental sampling was performed the day after patient discharge (these individuals were not in the vicinity when sampling was performed).

^d^ Two separate sampling events 6 days apart (i.e. visit 1 and visit 2) were performed around Case 5.

^e^ Cleaning was performed ca 24 hours before sampling and ca 6 hours before patient discharge.

^f^ Air vent not accessible; bathroom deposition sample collected instead.

^g^ No tap handle in patient room; sample from soap dispenser in bathroom collected instead.

^h^ The isolation room at this hospital has two anterooms (one for entry and one for exit). Samples were collected from both anterooms.

Blue shade: viral isolation attempted; blue shade and bold font: viral isolation attempted with successful identification of replicating virus.

Samples collected in the rooms of Cases 2, 3 and 4 frequently showed high Cq values (> 32.0), indicating low levels of MPXV DNA, or had no MPXV DNA detected (Table 2); however, all three cases had a severity score of ‘mild’ based on World Health Organization (WHO) guidance [4] (Table 1). In addition, all three cases were female and acquired mpox via household transmission which may contribute to the observed severity score; however, it is also important to note that these sampling events were performed post patient discharge which may also contribute to the lower levels of DNA observed. In contrast, more frequent detections of MPXV DNA, and instances of Cq value ≤ 32.0, were observed for Cases 1, 5 (both sampling events), and 8 (Table 2). These cases were either ‘moderate’ or ‘severe’ based on WHO severity score and the individual was present in the isolation room for all these sampling events. Interestingly, samples collected around Case 6 showed high Cq values or had no MPXV DNA detected (Table 2) despite this individual having ‘moderate’ clinical severity. This may be explained by this room being cleaned 12 hours prior to sampling as opposed to 24 hours for the remaining cases.

Virus isolation was attempted for a total of 21 samples containing detectable MPXV DNA, with four samples demonstrating presence of infectious virus (a light switch sample from the Case 1 isolation room, a tap handle sample from the ensuite bathroom for Case 5 during the first visit, the anteroom exit sample for Case 5 during the second visit, and the window ledge sample from the room of Case 8). Viral cultures derived from these four samples all had undetectable levels of MPXV DNA on day 0 of viral isolation with Cq values lower than 23.0 on day 7–10 of infection (data not shown). All positive viral isolations were from samples collected in isolation rooms with cases classed as either ‘moderate’ or ‘severe’ on the WHO severity score and were collected prior to patient discharge.

Of the air samples collected from the seven isolation rooms, only one had detectable MPXV DNA (the bed linen change sample from the second visit for Case 5); however, infection-competent virus was not identified in this sample.

## Discussion

The results from the currently reported investigations confirm that clade Ib mpox patients contaminate their immediate environment and that infection-competent virus may be present, which may pose a risk of onward transmission. While it is not possible to accurately quantify this risk using data from these investigations, they do support the need for defined infection prevention and control (IPC) measures when cases are detected to minimise the risk of exposure to viable virus in the environment that could present a transmission risk.

Findings from these environmental sampling investigations broadly align with those from studies performed in healthcare settings during the 2022 clade IIb public health emergency of international concern (PHEIC) [2,5-10]. Such information may contribute to the discussion regarding potential phenotypic differences between clade Ib and IIb MPXV; however, it is important to note the small sample size in both datasets. Our investigations again demonstrate that MPXV can be detected in air samples collected when bed linen is changed; however, detection of MPXV in air samples was uncommon in this study (only one of seven bed linen-change samples contained detectable MPXV DNA), despite the inclusion of some patients with high lesion-counts.

It is likely that the level of contamination seen in specific environments relates to several factors including clinical severity (e.g. the number of lesions), time spent in that environment, time since the patient was last present in the environment, and the frequency of cleaning of that environment. Monkeypox virus has notable environmental persistence, and infectious virus can survive numerous days on surfaces [11,12]. It is important to note that the isolation rooms concerned by our study were cleaned daily by healthcare workers with high touch points and the floors cleaned with commercially available products containing 5,000 ppm sodium hypochlorite; this frequent cleaning may contribute to our finding of only 4/21 samples containing infectious MPXV. Such IPC measures are relatively easy to adopt in controlled healthcare environments; however, less is understood about practical control measures in non-healthcare settings, such as domestic residences. Previous studies have demonstrated high levels of contamination in household environments [13-15], which probably occur due to a greater abundance of porous materials, combined with less frequent cleaning. Validation data are now available for disinfection in environments where chlorine-based solutions are less appropriate [16].

Limitations of this study include sampling events being conducted in rooms of patients with varying demographic characteristics, disease severity, and times since both onset and admission. In addition, two cases shared a room, which may have been contaminated in a different way than a single occupancy room (e.g. shared bathroom tap handle used more frequently) and two sampling events were performed post patient discharge. Most importantly, it is not possible to relate the extent of contamination identified, nor the presence of viable virus, to absolute transmission risk.

Additional data from environmental sampling around clade Ib mpox cases will assist in understanding how and when mpox patients contaminate their immediate environment and how this may affect the risk of transmission of infection in both healthcare and non-healthcare settings. It is also important to gain additional data relating to paediatric cases, in particular for cases of moderate or severe disease, due to the limited availability of detailed reports and the possible differences in how the importance of IPC measures is understood and applied. Such information can help inform proportionate IPC policies for specific environments; however, it is important to look at the totality of data on transmission risks and transmission events and view environmental sampling results alongside data from epidemiological studies, contact-tracing investigations, and detailed studies of contacts of cases that identify specific types of potential exposure and look closely for evidence of subsequent infection.

## Conclusion

This study confirms that people with clade Ib mpox can contaminate their immediate environment with MPXV, including replication-competent virus. Although the transmission risk via the environment cannot be accurately assessed, the findings underscore the importance of IPC measures and their continued adaptations to different epidemiological contexts.

## Data Availability

Not applicable.
